# Bilateral serous retinal detachment accompanied by a rare intraretinal fluid configuration in preeclampsia and PRES Syndrome

**Published:** 2019

**Authors:** Sibel Inan, Onur Polat, Ersan Cetinkaya, Umit Ubeyt Inan

**Affiliations:** * Afyonkarahisar Health Sciences University, Faculty of Medicine, Ophthalmology Department, Afyonkarahisar, Turkey; ** Afyonkarahisar State Hospital, Ophthalmology Clinic, Afyonkarahisar, Turkey; *** Antalya Education and Training Hospital, Antalya, Turkey; **** Afyonkarahisar Parkhayat Hospital, Ophthalmology Clinic, Afyonkarahisar, Turkey

**Keywords:** preeclampsia, posterior reversible encephalopathy syndrome, serous retinal detachment

## Abstract

**Purpose:** To present the association between posterior reversible encephalopathy (PRES) syndrome due to preeclampsia and bilateral serous retinal detachment (SRD) accompanied by intraretinal fluid configuration.

**Methods:** A 24-year-old woman, at 28 weeks of gestation presented with blurred vision bilaterally related to bilateral SRD involving the center of the macula accompanied by intraretinal fluid. The patient was diagnosed as pre-eclampsia accompanied by PRES syndrome. The patient approved and underwent delivery the same day. On day 9, ophthalmologic examination revealed complete resolution of SRD and normal visual acuity bilaterally and cranial MRI showed complete resolution of the vasogenic edema with medical treatment.

**Conclusion:** SRD and accompanying retinal edema must be considered among etiological factors leading to sudden vision loss in patients with preeclampsia and PRES syndrome.

**Abbreviations:** PRES = Posterior reversible encephalopathy, SRD = Serous retinal detachment, SD-OCT = Spectral-domain optical coherence tomography, RPE = Retinal pigment epithelium, CSC = Central serous chorioretinopathy, ONL = Outer nuclear layer, INL = Inner nuclear layer, IPL = Inner plexiform layer, RNFL = retinal nerve fiber layer

## Introduction

Posterior reversible encephalopathy (PRES) syndrome is a pathologic condition characterized by headache, visual disturbances, seizures and altered mental status. Treatment of the cause usually restores symptoms and neuroradiological findings within several weeks. If the condition is left untreated, however, cytotoxic edema and irreversible brain injury may occur [**1**].

Preeclampsia is a disorder characterized by high blood pressure, presence of protein in the urine, swelling and gaining excessive weight that occurs during pregnancy. Patients may complain of headache, visual disturbances, epigastric pain, or oliguria in addition to high blood pressure. Endothelial dysfunction, including vasoconstriction and end-organ ischemia is thought to play a major role in the pathogenesis of preeclampsia [**2**].

While a link between preeclampsia and serous retinal detachment (SRD) has previously been established, the association between PRES syndrome due to preeclampsia and bilateral SRD accompanied by intraretinal fluid configuration, to the best of our knowledge, has not been reported yet. 

## Case report

**Fig. 1:**
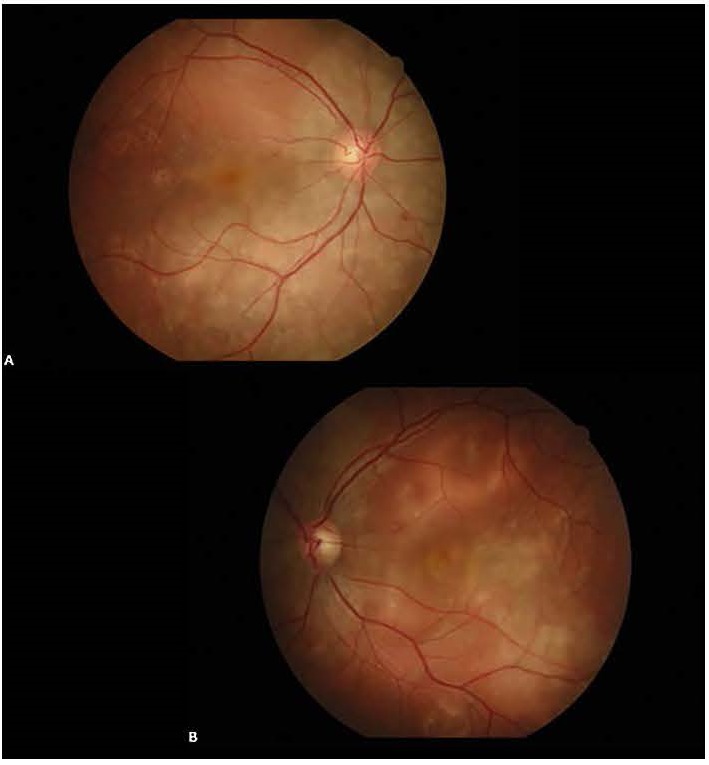
Bilateral retinal detachments affecting the posterior pole; at the time of presentation, right eye (A), left eye (B)

A 24-year-old pregnant woman, at 28-weeks’ gestation, presented with bilateral visual impairment that started one day before. Physical examination and laboratory studies revealed hypertension (170/100mmHg), proteinuria (100mg/ dl), elevated transaminases and decreased platelet count. Visual acuity was 20/ 100 with -1.00-0.50x4 correction in the right; and 20/ 50 with -1.00+0.50x71 correction in the left eye. Intraocular pressure was 14 mmHg bilaterally. While the anterior segment examination was normal, fundus examination showed bilateral retinal detachments affecting the posterior pole (**Fig. 1**).

**Fig. 2:**
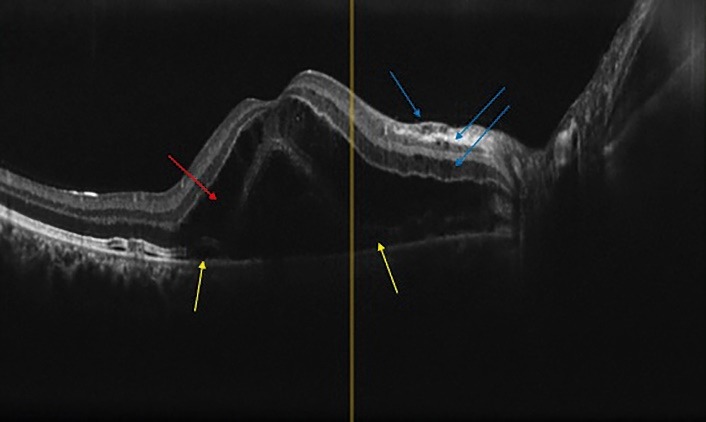
OCT of right eye. Cystic fluid accumulation is seen between the separating septae throughout the ONL (red arrow). Some small cysts are also present in INL, IPL and RNFL (blue arrow). A very shallow elevation of interdigitation zone in both side of foveal center is also apparent (shallow SRF, yellow arrows)

**Fig. 3:**
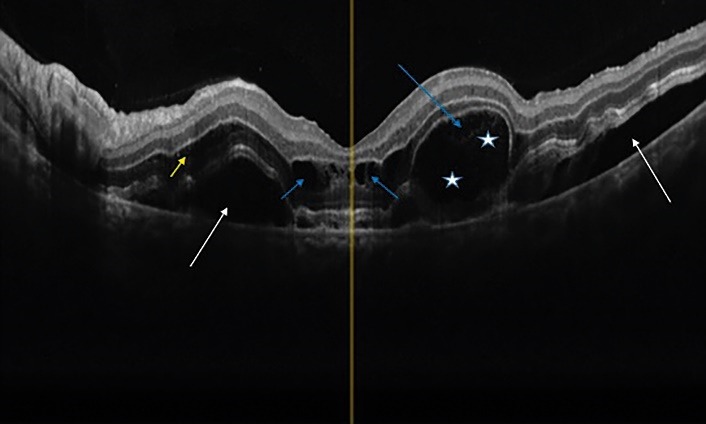
OCT of left eye, cystoid macular edema with several cysts (big and little mixed together) in the Henle Layer just beneath the foveal center (blue arrows). Split like separation between OPL and ONL possible due to fluid accumulation is also apparent at the nasal side of foveal center (yellow arrow). A truly sub-retinal fluid is seen at both the nasal and temporal side of fovea (white arrows). An interesting OCT configuration is seen just at temporal side of foveal center with very large detachment of ellipsoid zone and development of a large cystoid cavity containing serous and medium dense fluid (purple arrow, white stars)

Spectral-domain optical coherence tomography (SD-OCT) demonstrated bilateral SRD involving the center of the macula accompanied by intraretinal fluid (**Fig. 2**,**3**). 

**Fig. 4:**
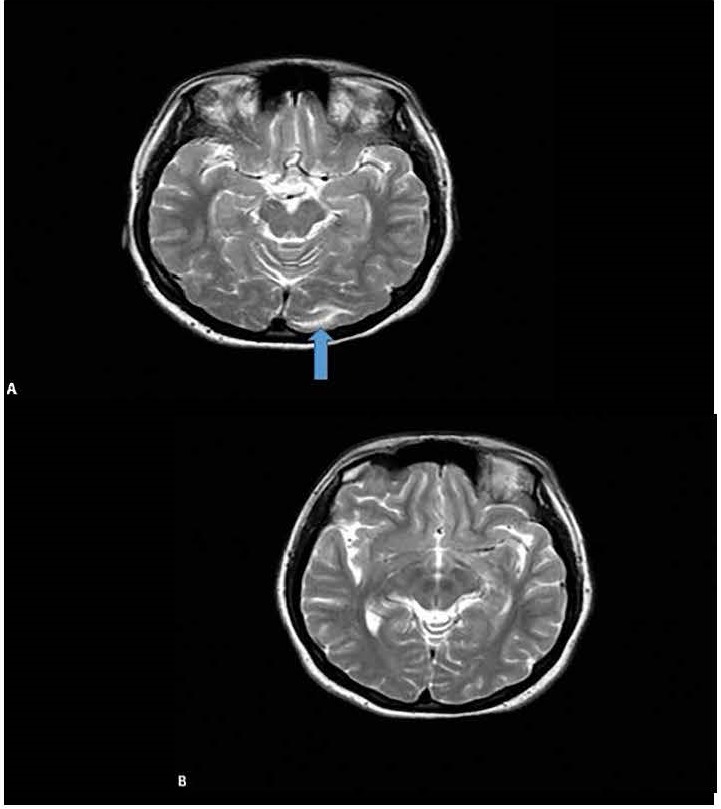
MRI; hyperintense vasogenic edema in the white matter of the left occipital lobe (blue arrow) (A); resolution of vasogenic edema at day 9 (B)

**Fig. 5:**
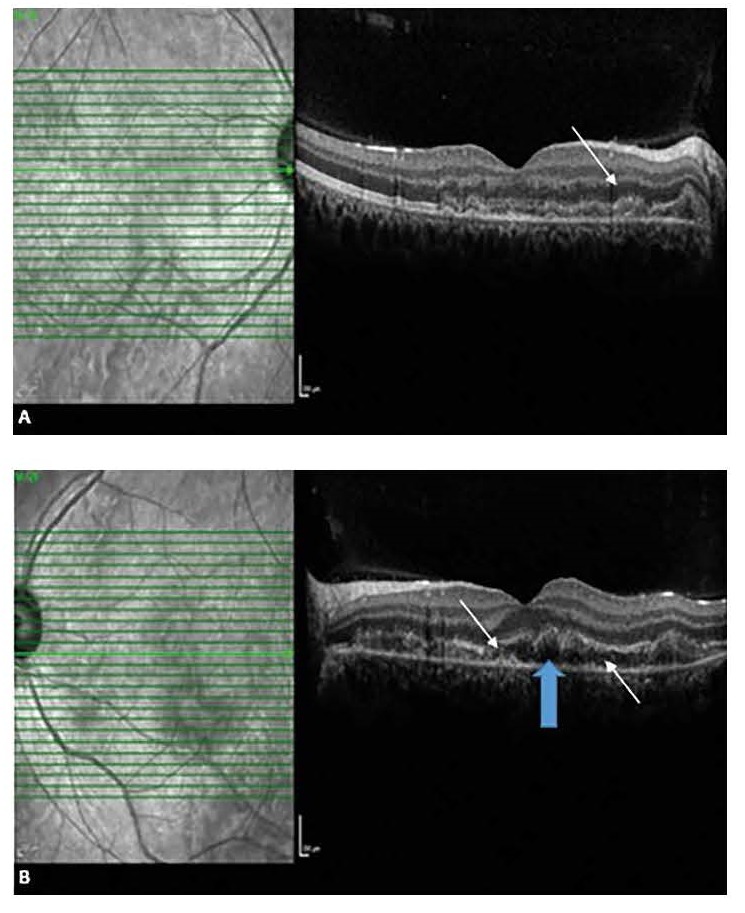
Intraretinal fluid resolved completely and SRD resolved partially in the right (A) and left eye (B) on day 4. Swelling in the outer most retinal layers with very shallow sub retinal fluid is seen (blue arrow). Many hyper reflective dots predominately in the outer retinal layers are also present (white arrows)

**Fig. 6:**
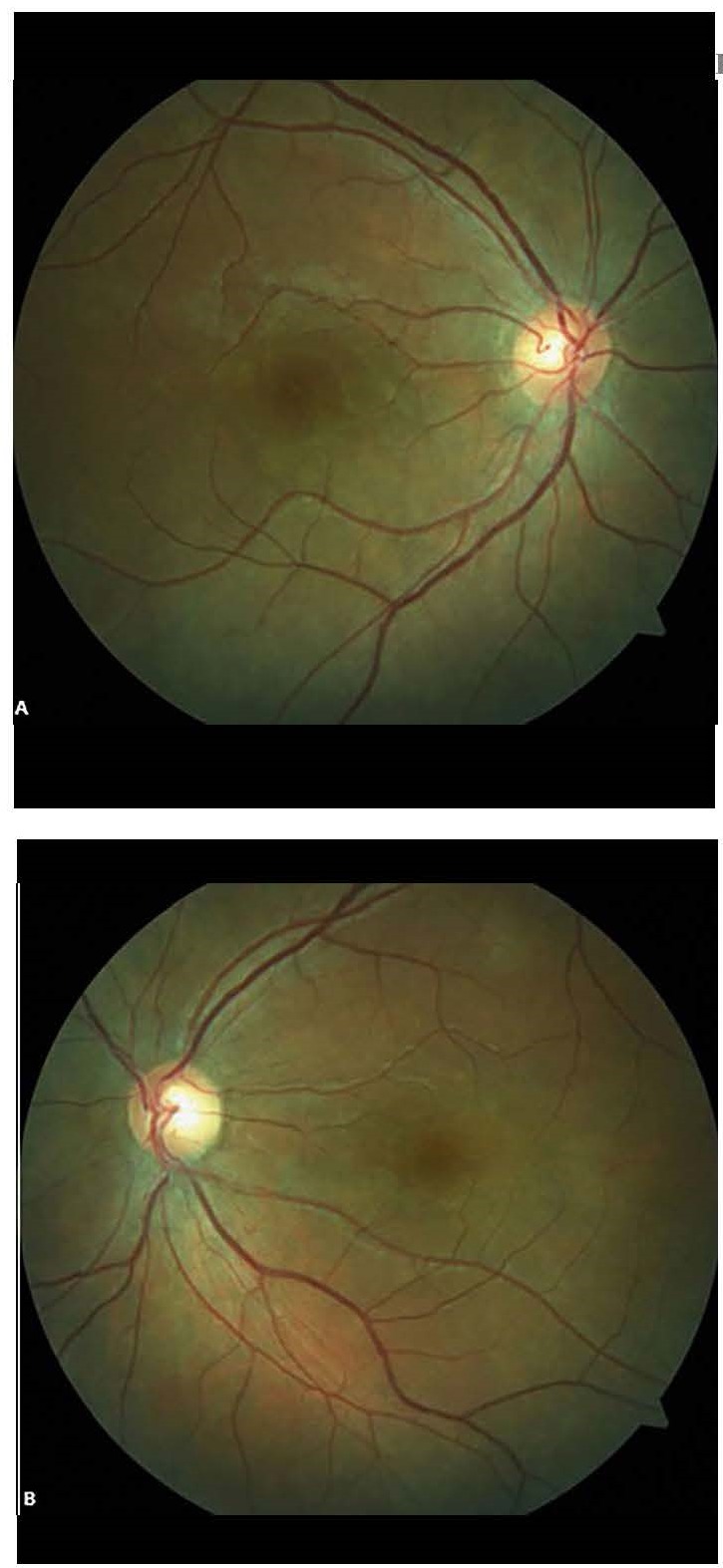
Retinal detachment was completely resolved at day 9, right eye (A), left eye (B)

**Fig. 7:**
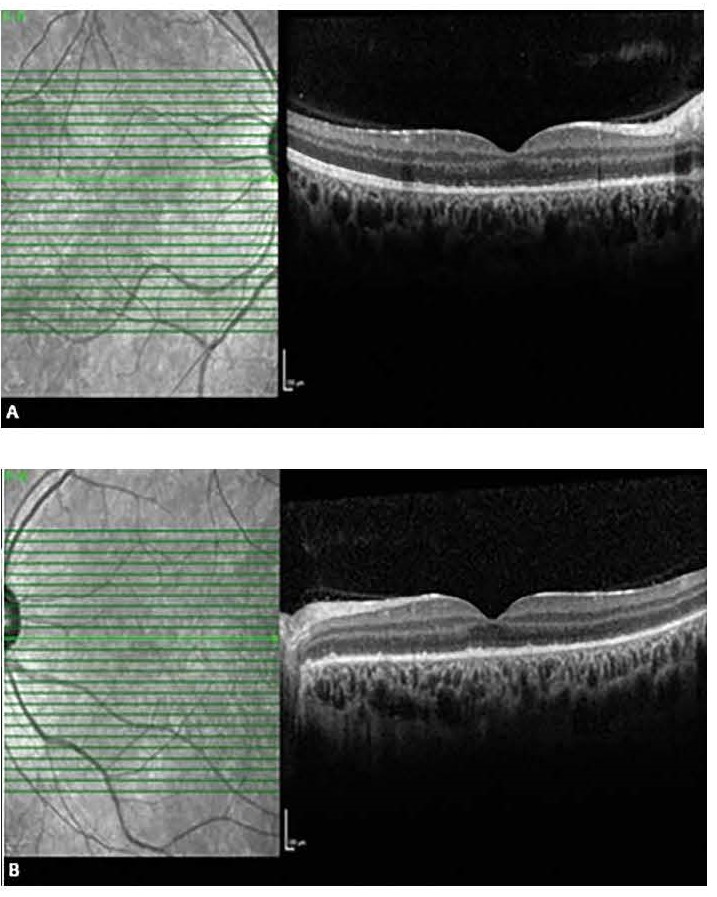
SRD resolved completely on day 9. All retinal layers appeared normal with complete disappearance of hyper reflective dots, right eye (A), left eye (B)

The patient underwent cesarean section the same day by obstetrician with patient approval. Following delivery, nifedipine 30mg po bid was commenced due to high blood pressure (150/ 80 mmHg). Due to the depressed consciousness of the patient, MRI was performed and T2-FLAIR sequences demonstrated hyperintense vasogenic edema, particularly, the white matter of the left occipital lobe (**Fig. 4A**). On day 4 while confusion persisted, SRD resolved (**Fig. 5**) and visual acuity improved to 20/ 25 bilaterally. On day 9 cranial MRI showed complete resolution of the vasogenic edema of the left occipital lobe (**Fig. 4B**). A repeated ophthalmoscopy revealed complete resolution of SRD and normal visual acuity, bilaterally (**Fig. 6**,**7**). At 6-weeks after delivery, blood pressure was 110/ 60mmHg, and the patient fully recovered. A final ophthalmologic examination was unremarkable and showed normal outcomes in terms of visual acuity, intraocular pressure, pupillary light reflex, anterior segment, and fundus examinations. SD-OCT revealed attached macula. The patient did not accept further etiopathological studies with FFA and ICG at the presentation and after the delivery.

## Discussion

 The etiology of PRES syndrome includes hypertensive encephalopathy, preeclampsia, eclampsia, cyclosporine-A neurotoxicity, acute glomerulonephritis and thrombotic thrombocytopenic purpura [**1**]. Preeclampsia and eclampsia account for 7 to 20% of all PRES syndrome cases [**1**,**3**]. Diagnosis is established by MRI and the imaging appearance is typical and involves bilateral symmetric hyperintensity within the white matter of the parieto-occipital regions suggestive of vasogenic edema [**4**]. In the current case, we observed that the hyperintense vasogenic edema within the left occipital lobe had completely disappeared on the MRI control on day 9. 

PRES syndrome may be associated with a wide variety of visual disturbances such as cortical blindness, visual neglect, homonymous hemianopia, and blurred vision. Association rates up to 34% have been reported [**5**]. It is well known that pregnancy and/ or hypertension is a risk factor for the development of SRD. Subretinal fibrinous exudates are also observed in such cases [**6**]. Numerous studies have previously suggested an association between hypertension and choroidopathy [**7**]. Briefly, vasoconstriction of choroidal arterioles is believed to account for the choroidopathy observed in the setting of hypertension. Histopathologic studies provided the evidence and revealed constriction and fibrinoid necrosis of choroidal arterioles in patients who died due to conditions related to hypertensive emergencies [**8**].

Although retinal detachment has been reported in preeclampsia, intraretinal cystic fluid accumulation as seen in our case is not a known entity. It is not known whether these cystic cavities represent a truly fluid accumulation or empty cavities possibly or speculatively due to the presence of a sticky subretinal fibrin and the outer retina adherent to the retinal pigment epithelium (RPE) in these points. However, we believe that macular edema may occur due to a leakage of fluid across the RPE secondary to choroidal vasculopathy and, in part, due to RPE pump dysfunction. Intraocular inflammation may also be another contributory factor in the development of intraretinal cystic edema in our case.

Some of the most striking associations with SRD in the current case include the following: RPE detachment observed on OCT, intraretinal cystoid edema, fluid collection within ellipsoid and RPE layers and numerous hyperreflective foci on the outer retinal layers. Thorsrud et al. [**9**] presented a preeclamptic woman accompanied by combined central serous chorioretinopathy (CSC) and PRES syndrome. However, their case did not include such diversified findings in OCT examination such as RPE detachment and intraretinal changes as observed in our case. They also called retinal involvement as CSC in their case. Additionally, visual acuity of light perception in both eyes, despite no foveal involvement in the right eye at presentation, implies a cortical blindness in their case [**10**].

In conclusion, SRD may be accompanied by RPE detachment and intraretinal cystic changes in preeclamptic pregnant. Serous retinal detachment is usually resolved after the delivery. Although accompanying PRES syndrome is also usually reversible, prompt diagnosis and treatment is essential to prevent related complications.

**Disclosure Statement**

The authors declared no conflict of interest and this study received no financial support.
